# Circular RNAs: New Players in Cardiomyopathy

**DOI:** 10.3390/genes13091537

**Published:** 2022-08-26

**Authors:** Maedeh Bagheri Moghaddam, Majid Maleki, Maziar Oveisee, Mahrokh Bagheri Moghaddam, Maedeh Arabian, Mahshid Malakootian

**Affiliations:** 1 Molecular Genetics Department, Faculty of Biological Sciences, Tarbiat Modares University, Tehran 141171311, Iran; 2 Cardiogenetic Research Center, Rajaie Cardiovascular Medical and Research Center, Iran University of Medical Sciences, Tehran 1995614331, Iran; 3 School of Medicine, Bam University of Medical Sciences, Bam 7661771967, Iran

**Keywords:** circular RNAs, cardiomyopathy, non-coding RNAs, ceRNA

## Abstract

Cardiomyopathies comprise a heterogeneous group of cardiac diseases identified by myocardium disorders and diminished cardiac function. They often lead to heart failure or heart transplantation and constitute one of the principal causes of morbidity and mortality worldwide. Circular RNAs (circRNAs) are a novel type of noncoding RNAs. They are covalently closed and single-stranded and derived from the exons and introns of genes by alternative splicing. This specific structure renders them resistant to exonuclease digestion. Many recent studies have demonstrated that circRNAs are highly abundant and conserved and can play central roles in biological functions such as microRNA (miRNA) sponging, splicing, and transcription regulation. Emerging evidence indicates that circRNAs can play significant roles in cardiovascular diseases, including cardiomyopathies. In this review, we briefly describe the current understanding regarding the classification, nomenclature, characteristics, and function of circRNAs and report recent significant findings concerning the roles of circRNAs in cardiomyopathies. Furthermore, we discuss the clinical application potential of circRNAs as the therapeutic targets and diagnostic biomarkers of cardiomyopathies.

## 1. Background

### 1.1. Cardiomyopathies

The American Heart Association (AHA) in 2006 defined cardiomyopathies as a heterogeneous group of disorders of the myocardium that can change cardiac function (mechanical and/or electrical dysfunction) and structure and lead to heart failure and cardiovascular death [1,2,3,4]. Cardiomyopathies can be categorized into two main groups according to the prevailing organ involvement and the evolution of genetic testing and diagnostic imaging methods in cardiology [5,6]. Primary cardiomyopathies, which are caused by genetic, nongenetic, and acquired conditions, consist of dilated cardiomyopathy (DCM), hypertrophic cardiomyopathy (HCM), restrictive cardiomyopathy (RCM), and arrhythmogenic cardiomyopathy (ACM). Secondary cardiomyopathies comprise a group in which the pathological myocardial disease is the outcome of a systemic (multiorgan) condition [1]. In 2008, the European Society of Cardiology updated the classification system for cardiomyopathy. It classified patients based on morphological and functional phenotypes. In this classification, the use of the terms primary and secondary cardiomyopathy were not applied for cardiomyopathies [4,7]. In 2013, the MOGE(S) classification for cardiomyopathy was proposed by Arbustini et al. [8]. In this classification, which was endorsed by the World Heart Federation, M refers to the phenotype, O refers to organ involvement, G refers to genetic transmission, E refers to pathogenesis, and S refers to disease stage.

### 1.2. Noncoding RNAs (ncRNAs)

**Figure 1:**
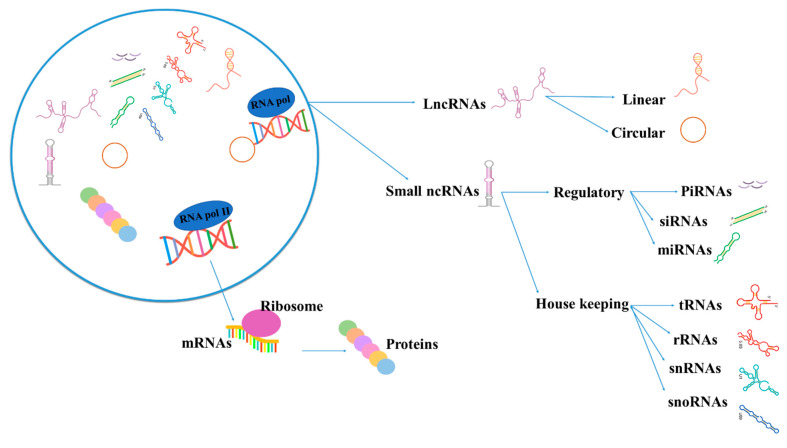
The image depicts the classification of ncRNAs: rRNA, ribosomal RNA; tRNA, transfer RNA; snRNA, small nuclear RNA; snoRNA, small nucleolar RNA; miRNA, microRNA; siRNA, small interfering RNA; PiRNA, Piwi-interacting RNA; lncRNA, long noncoding RNA.

Accumulating evidence indicates that a great part of the genome is transcribed. Nonetheless, while only a small percentage of the genome encodes proteins, most of it encodes ncRNAs, which theoretically do not encode proteins [9,10,11]. Recent studies have shown that ncRNAs function as molecular regulators and have a momentous functional role in cellular homeostasis and disease pathophysiology [12,13,14,15]. Therefore, ncRNAs form a very heterogeneous group of RNAs divided into small (<200 nt in length) and long (>200 nt in length) ncRNAs based on their size. In this regard, Piwi-interacting RNAs (piRNAs), microRNAs (miRNAs), and small interfering RNAs (siRNAs) are categorized into the small ncRNA group, and circular RNAs (circRNAs) and long noncoding RNAs (lncRNAs) are characterized into the lncRNA group (Figure 1). Additionally, based on their function, ncRNAs can be divided into housekeeping and regulatory ones. Housekeeping ncRNAs, composed of ribosomal RNAs (rRNAs), transfer RNAs (tRNAs), small nuclear RNAs (snRNAs), and small nucleolar RNAs (snoRNAs), are expressed in all cell types and perform crucial functions in cells, whereas regulatory ncRNAs, consisting of miRNAs, circRNAs, and lncRNAs, cooperate in the regulation of gene expression [16,17,18,19].

### 1.3. CircRNAs

#### 1.3.1. Formation (Biogenesis) and Classes of circRNAs

CircRNA, a type of ncRNA, constitutes a group of single-stranded RNAs covalently forming a closed-loop framework without the usual terminal structures of RNAs (5′ cap or a polyadenylated tail). A special alternative splicing mode termed “backsplicing”, which does not follow the same canonical 5′–3′ polarity, is responsible for generating circRNAs. A closed structure is formed via the backsplicing of pre-messenger RNAs (premRNAs) by the ligation of the 3′ end of an exon to the 5′ end of its own or an upstream exon via a 3′,5′-phosphodiester bond [20,21,22]. In general, circRNAs are catalyzed either by the spliceosomal machinery or by ribozymes (Group I and Group II) [23]. Two models of circRNA biogenesis, the lariat model and the direct backsplicing model, have been determined and validated [21,24,25]. Li et al. [26] recently demonstrated that the assembling of the spliceosome E complex on premRNAs could cross an exon in which it either remodeled to span an intron for canonical linear splicing (typically on short exons) or drove backsplicing to make circRNAs (on long exons).

CircRNAs may originate from exons or introns, culminating in the development of three different types of circRNAs: exonic (ecircRNAs), intronic (ciRNAs), and exon-intron (elciRNAs) [25]. 

EcircRNAs comprise a notable proportion of the discovered circRNAs. They are linear transcripts without introns and are mostly present in the cytoplasm. This type of circRNA is formed via two model mechanisms. The first one is the lariat-driven circularization model, in which the 3′ splice site of the acceptor is joined with the 5′ splice site of donor exons; then, the intron between these exons is eliminated, and the exons form a lariat. The second one is the intron pairing-driven circularizing model, formed based on reverse complementary matches (RCMs) within flanking introns. In detail, base pairing between flanking introns is induced by RCMs following the formation of hairpins. Hairpin formation brings the 5′ and 3′ termini of an exon into spatial proximity, resulting in “head-to-tail” splicing. In this mechanism, adenosine deaminases acting on RNAs (ADARs) are involved, together with RCMs [27]. Moreover, a corresponding elevation in the number of circRNAs has been noted, correlating with the number of exons per gene [28]. 

CiRNAs are intronic RNAs without exonic sequences. This type of circRNA is not developed via backsplicing. Additionally, ciRNAs have the limited enrichment target site of miRNAs, exist mostly in the nucleus, and regulate the expression of their parental genes. The 7 nt GU-rich sequence, close to the 5′ splicing site, and the 11 nt C-rich motif, close to the 3′ branchpoint site, play essential roles in the formation of ciRNAs [25,29,30,31,32].

ElciRNAs contain both introns and exons in their sequences and boost the transcription of their parental genes through interactions with U1 small nuclear ribonucleoprotein particles (snRNPs) and pol II. They are mostly localized in the nucleus; nevertheless, the mechanism of elciRNA formation is still unknown. The production of elciRNAs could be facilitated through premRNAs, encompassing flanking Alu complementary pairs or flanking complementary sequence pairs other than Alu [25,29,33]. 

**Figure 2:**
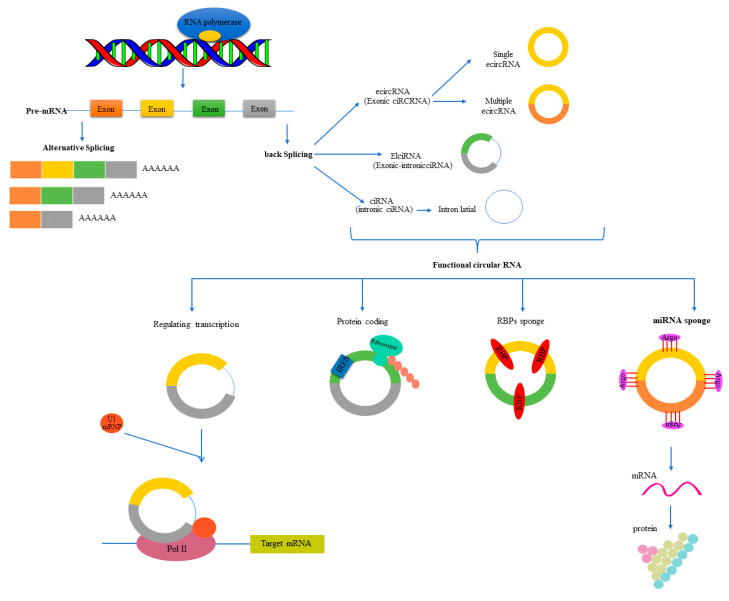
The image illustrates the classification and function of circRNAs.

Furthermore, corresponding to their genomic location, circRNAs can be divided into two groups: intergenic and intragenic. The intergenic group consists of non-exonic circRNAs harbored between two genes, while the intragenic group is located in genes (Figure 2) [34]. 

#### 1.3.2. Nomenclature of circRNAs

Despite numerous circRNA studies, there is no standard nomenclature for circRNAs. Recently, the circBank database introduced a novel naming system for circRNAs based on the host gene of the circRNA and the starting/ending location of the circRNA in the host gene. According to the circBank, human circRNAs are named depending on the Human Genome Organization (HUGO) host gene symbol, shown by the following scheme: “*hsa-circHUGO-#*”. Further, circRNAs emanating from the same host genes are numbered according to their respective location in the host gene, with the upstream one allocated as the starting number. If circRNAs start in the same starting site and end in a different ending site, the earlier ending site is assigned the lower number. For circRNAs with the same starting site and the same ending site, the alternative splicing of the circRNA is considered. In this respect, the circRNA nomenclature includes “*hsa-circHUGO-#_V#*”, in which “*V*” stands for “variant”, and the number after *“V”* depends on the length of the circRNA. The shorter circRNA is earmarked the earlier number. 

For the nomenclature of intergenic circRNAs, the “*hsa-circChrom#_#*” scheme is applied, whereby the first number denotes the chromosome number, and the circRNA order number is placed following the same rule as that for circRNA form-coding genes [35].

#### 1.3.3. Localization of circRNAs

Advances in high-throughput sequencing and in vivo and in vitro experimental validation and bioinformatics have confirmed the existence of circRNAs as a separate class of ncRNAs that can be enriched in the cytoplasm, the nucleus, the mitochondria of the cells, and body fluids, including whole blood, plasma, serum, saliva, seminal fluid, and urine [36,37,38,39].

Exonic circRNAs are mostly localized in the cytoplasm [37], although some of them are detected in the nucleus, where they are chiefly involved in the augmentation of the nuclear retention of proteins or delivery of proteins to chromatin [40,41]. Some studies have demonstrated that ciRNAs are mostly retained in the nucleus and are involved in the regulation of parental gene expression [30,33]. Evidence also indicates that some circRNAs are located in mitochondria [42,43].

#### 1.3.4. Functions of circRNAs

There is a growing body of evidence demonstrating that circRNAs function as the molecular regulators of gene expression at the level of transcription and post-transcription in the nucleus and the cytoplasm. CircRNAs regulate the expression of the target by acting as sponging miRNAs, holding RNA-binding proteins (RBPs), translating RNA into polypeptides, and controlling the alternative splicing of their parental gene (Figure 2) [20,21,31,44,45]. 

Many studies have posited that circRNAs might have serious roles in the cause, development, and progression of human diseases, including central nervous system diseases [46,47] and various cancers such as lung cancer [48], osteosarcoma [49], renal cancer [50], hepatocellular carcinoma [41], gallbladder cancer [51], and breast cancer [52].

Recent research shows that the profile expression of circRNAs is associated with different types of cardiovascular diseases such as cardiomyopathies, chronic heart failure, and coronary artery disease [53,54,55,56,57,58,59]. Jakobi et al. [60] reported that circRNAs generated from *Hectd1*, *Ppp2r3a*, *Slc8a1*, *Dmd*, and *Ttn* host genes were associated with cardiomyopathies.

In addition, perturbation in RNA editing can affect the secondary structure of RNAs, regulate circRNA formation, and thus cause human diseases [25,61]. Previous investigations of the transcriptome sequencing of the myocardium demonstrated that adenosine-to-inosine (A-to-I) RNA editing underlays 80% of editing events. A reduction in RNA editing is one of the characterizations of failing human hearts and is attributed to Alu elements in the introns of protein-coding genes [25,61]. In a study on the expression profile of failing left ventricle, 166 circRNAs were upregulated and 7 circRNAs were downregulated compared with non-failing ones. The results of that study also showed that a reduction in RNA editing in the host gene was associated with the majority of upregulated circRNAs [61]. 

In vivo studies have demonstrated that some circRNAs can be translated [62,63,64,65]. Several studies have revealed that circRNAs have a longer half-life and are more resistant to ribonuclease R (RNAse R) than other ncRNAs due to their unique structure. Consistent with these findings, circRNAs can be promising biomarkers and therapeutic targets for diseases [36,63,66,67]. 

The following section discusses the roles of circRNAs in different types of cardiomyopathies.

## 2. CircRNAs in Cardiomyopathies

### 2.1. DCM

DCM is a type of nonischemic cardiomyopathy characterized by left or biventricular dilation and decreased systolic function. DCM causes can be classified as genetic and nongenetic, with a great number of genes and alleles involved in its pathogenesis. The exact genetic DCM prevalence has yet to be determined. A detectable genetic cause has been reported in 40% of familial DCM cases, and pathogenic genetic variants have been detected in sporadic DCM [68,69].

Several studies have demonstrated that ncRNAs, including lncRNAs and miRNAs, play key roles in DCM. For instance, lncRNA H19 boosts cardiomyocyte apoptosis in patients with DCM [70]. Additionally, circulating lncRNA ENST00000507296 is a probable prognostic biomarker [71], and circulating miR-3135b, miR-3908, and miR-5571-5p might be considered diagnostic biomarkers of DCM [72,73].

#### 2.1.1. CircSLC8A1 

CircSLC8A1 is one of the most abundant circRNAs in CMs arising from the second exon, with a length of 1832 bp of the sodium–calcium exchanger gene *Slc8a1*, known to be involved in arrhythmias [74,75]. CircSLC8A1 was discovered by Li et al. [74] in 1999 (in the “pre-NGS era”). The authors were the first to report that this circRNA coded a truncated protein with the ability of Na/Ca exchange in HEK cells. Recently, it has been demonstrated that circSLC8A1 is mainly located in the cytoplasm of cells and functions as an endogenous miRNA sponge to regulate the expression of genes [28,76]. In addition, circSlc8a1 has an essential role in CM differentiation, cardiac development, and homeostasis. Thus, dysregulation in the expression of this circRNA might contribute to heart disorders [75,77,78,79,80].

A previous study demonstrated that the highly specific expression of circSLC8A1, along with another five circRNAs (SLC8A1, ARID1A, FNDC3B, CACNA1D, SPHKAP, and ALPK2), emanated from the exons of protein-coding genes in human-induced pluripotent stem cell (hiPSC)-derived CMs, while circAASS, circFIRRE, and circTMEFF1 expression levels were sharply downregulated in hiPSC-derived CM fibroblasts. Therefore, the cardiac-specific expression of circSLC8A1, circCACNA1D, circSPHKAP, and circALPK2 circRNAs indicated the potential role of these RNAs as biomarkers of CMs [79]. In detail, high expression levels of circSLC8A1, circCACNA1D, and circSPHKAP RNAs were detected on days 9, 15, and 30 of cardiac differentiation in beating CMs. The abundant expression of circALPK2 was found in cells on day 4 of cardiac differentiation, and regardless of the expression of circFNDC3B transcripts in all stages of differentiation, the expression of these circular transcripts was raised considerably in differentiated CMs from day 9 [79].

A prior investigation showed the expression of circSLC8A1_11 and circ-SLC8A1_12, generated from *SLC8A1* in the normal heart and concluded that it was involved in the maintenance of cardiac homeostasis [81]. 

Moreover, the expression of circSLC8A1 is upregulated in the heart tissues of patients with DCM compared with control groups, and a positive correlation exists between circSLC8A1 expression and its linear isoform, whereas circSLC8A1 expression is more stable and much higher than that of the other transcripts of SLC8A1 [79,80].

#### 2.1.2. Deregulated circRNAs in DCM

Accumulative research has introduced different circRNAs mostly originating from genes whose mutations cause DCM. The remarkable misexpression of circRNAs generated from *CHD7*, *ATXN10*, and *DNAJ6C* was found in patients with DCM in comparison with a control group in a previous investigation. There was an upregulation in circ-CHD7 and circ-ATXN10, while the expression of circ-DNA6JC was downregulated. The study suggested novel therapeutic targets given the new signatures of potential disease-relevant circRNAs [80].

The results of the analysis of RNA high-throughput sequencing on the heart samples of patients with DCM revealed 9585 circRNAs, with differential expression levels. Of this total, 213 circRNAs were upregulated and 85 were downregulated. The top 10 upregulated circRNAs were generated from *ICA1*, *TTN*, *BTBD7*, *FAT1*, *LYPLAL1*, *NHLRC2*, *DHX40*, and *PKN2* genes, all of which except circ-LYPLAL1 and circ-NHLRC2 (sense-overlapping circRNAs) were exonic circRNAs. Further, *MYH7*, *EBF1*, *ZNF670*, *SEC23A*, *NBEA*, *TTN*, *PDE1C*, *CTNND2*, *ATRX*, and *OR2A1-AS1* genes generated the top 10 downregulated circRNAs. In addition, circRNAs from *EBF1*, *SEC23A*, *NBEA*, *PDE1C*, *ATRX*, and *OR2A1-AS1* were exonic, whereas circ-ZNF670 and circ-CTNND2 were intronic circRNAs. The results of that investigation also demonstrated that *MYH7* and *TTN* genes developed sense-overlapping circRNAs. Quantitative reverse transcription-polymerase chain reaction (qRT-PCR) confirmed the upregulation of circRNAs from *ICA1, FAT1*, and *LYPLAL1*, as well as the downregulation of circ-EBF1, circ-ZNF670, and circ-NBEA [82]. 

Dong et al. [81] reported a list of circRNAs enriched in normal and DCM hearts based on an RNA-seq data-set analysis of left ventricular tissues of five patients with DCM and five healthy controls. Their analysis showed that *NPPA* expression was highly increased in the DCM samples, as well as in the circRNAs derived from *MYH6* and *MYH7* genes, which are highly enriched and conserved in the heart of humans, mice, and rats. Since these two genes have crucial functions in healthy hearts and cardiovascular diseases, their circRNAs are important and have roles in the pathophysiology of heart diseases, including DCM. 

The largest number of exons in the human genome belongs to *Titin* (*TTN*) and *Ryanodine receptor 2* (*RYR2*) genes, which produce 197 and 173 circRNAs, respectively. Some circRNAs of these two genes, including circTTN_34, circTTN_52, circTTN_70, circTTN_132, circRYR2_71, and circRYR2_95, are downregulated in DCM [81]. 

Read-through circRNAs (rt-circRNAs) are a newly discovered type of circRNAs generated from two neighbor genes on the same strand. Most rt-circRNAs originate from *SCAF8* and *TIAM2* genes, which are dysregulated in DCM. They can also sponge several miRNAs linked to heart diseases, exemplifying this phenomenon. Moreover, SCAF8_e4: TIAM2_e1, and SCAF8_e4: TIAM2_e2 are considerably downregulated in DCM [81].

One of the causes of DCM is the mutation in the *RNA-binding motif protein 20 (RBM20)* gene [83], which is vital for the appropriate splicing of a great number of genes. In addition, RBM20 is critical for the organization of a subclass of circRNAs derived from a specific region within the TTN I-band [84]. The loss of function of RBM20 leads to defects in the splicing of the *TTN* gene [85,86], as well as the development of a specific circRNA TTN subclass involved in the pathophysiology of DCM (Table 1) [84].

### 2.2. HCM

HCM, described in the 1950s for the first time, is one of the most prevalent inherited and heterogeneous cardiomyopathies [87,88,89,90]. HCM prevalence is estimated at 1 in 500 people, although recent investigations have reported an even higher prevalence rate [91,92]. 

Recently, three circRNAs (circDNAJC6, circMBOAT2, and circTMEM56) have been implicated in HCM. According to a prior study, the expression levels of these three circRNAs were significantly decreased in the serum samples of patients with HCM compared with a healthy group. Further, a negative correlation existed between the severity of left ventricular obstruction and the thickness of the interventricular septum and the expression levels of two circRNAs (TMEM56 and DNAJC6) [93].

Six circRNAs (hsa_circ_0011555, hsa_circ_0036248, hsa_circ_0041499, hsa_circ_0041554, hsa_circ_0043762, and hsa_circ_0071269) were introduced as RNAs related to HCM by Guo et al. [94], who performed a circRNA microarray assay on plasma samples from 15 patients with HCM and 7 healthy controls.

Another study reported that mm9-circ-012559, a heart-related circRNA (HRCR), was downregulated in a mouse model of failing hearts. The results also demonstrated that circ-HRCR acted as an anti-hypertrophic molecule causing the upregulation of ARC expression by sponging miR-223, which is related to the progression of cardiac hypertrophy and heart failure [95].

Guo et al. [94] conducted a circRNA microarray assay using plasma samples from 15 patients with HCM and 7 controls. They found that hsa_circ_0011555, hsa_circ_0036248, hsa_circ_0041499, hsa_circ_0041554, hsa_circ_0043762, and hsa_circ_0071269 were correlated with HCM. Their gene ontology (GO) analysis demonstrated that hsa_circ_0071269 and hsa_circ_0043762 were enriched during the activity of the calcium-release channel. Conversely, hsa_circ_0036248 was embellished during the activity of the calcium-release channel and the sliding of muscle filaments. In addition, the results of the KEGG analysis demonstrated that hsa_circ_0036248 might regulate transient receptor potential (TRP) channels, adrenergic signaling in CMs, and calcium signaling pathways. The authors concluded that since the expression of TRP channels increased in the HCM model and contributed to diastolic calcium overload, hsa_circ_0036248 might be involved in HCM, while hsa_circ_0071269 was associated with DCM through the regulation of TRP channels (Table 2). 

## 3. Diabetic Cardiomyopathy

Diabetic complications are the principal cause of death in patients with diabetes [96]. The phenomenon is exemplified by such cardiovascular problems as diabetic cardiomyopathy, which accounts for 80% of diabetic deaths [97]. The term “diabetic cardiomyopathy” was introduced four decades ago by Rubler [98], who reported the death of four patients with diabetes mellitus due to heart failure. Diabetic cardiomyopathy is the main cause of morbidity and mortality, the prevalence of which is positively correlated with the incidence of obesity, type II diabetes mellitus, insulin resistance, and hyperinsulinemia in developed countries [99]. Moreover, diabetic cardiomyopathy occurs in patients with type I or type II diabetes mellitus regardless of hypertension or other cardiovascular diseases [100].

### 3.1. Circ-HIPK3

CircHIPK3 is an oncogene circRNA originating from the second exon of *homeodomain-interacting protein kinase 3* (*HIPK3*), usually localized in the cytoplasm of cells [101,102].

In a previous study, Circ-HIPK3 was upregulated in the ventricular tissues of diabetic mice. The knockdown of circ-HIPK3 decreased fibrosis in myocardial tissue and enhanced left ventricular function in a mice model of diabetic cardiomyopathy. Furthermore, circ-HIPK3 enhances the synthesis of types I and III collagen by acting as a competing endogenous RNA (ceRNA), sponging miR-29b-3p, and upregulating the expression of COL1A1 and COL3A1 [103].

### 3.2. Dysregulated circRNAs 

Yang et al. [104] found that the expression level of hsa_circ_0076631, a novel circRNA named “caspase-1-associated circRNA (CACR)”, was highly increased in high-glucose-treated CMs and the serum of diabetic patients. CACR is localized in both the nucleus and the cytoplasm of the cell and regulates the pyroptosis and expression of caspase-1 by playing the role of a ceRNA and sponging miR-214-3p. The authors suggested that CACR could act as a clinical biomarker of diabetic cardiomyopathy and might be a new therapeutic target for diabetic cardiomyopathy, because silencing CACR could exert cardioprotective effects by significantly repressing CM pyroptosis, inflammation, and death. 

CircRNA_000203 is upregulated in the myocardium of diabetic mice, as well as in the cardiac fibroblasts of Ang-II-induced mice. This circRNA is generated from exon 7 to exon 15 of *Myo9a* as a host gene, and it causes the upregulation of Col1a2, Col3a1, and α-SMA expression in cardiac fibroblasts. In detail, the inhibitory effect of miR-26b-5p on Col1a2 and CTGF targets is suppressed through the sponging of miR-26b-5p by circRNA_000203, resulting in increased Col1a2 and CTGF expression levels. This circRNA is proposed as a potential target for the prevention and treatment of cardiac fibrosis in diabetic cardiomyopathy [105].

The expression of circRNA_010567 exhibits a significant rise in the myocardium of diabetic mice and cardiac fibroblasts treated with Ang II. Additionally, knocking down the expression of circRNA_010567 culminates in repressing the expression of Col I, Col III, and α-SMA, which is associated with fibrosis in cardiac fibroblasts, and upregulating miR-141, which leads to the downregulated expression of TGF-β1 [106].

Dong et al. [107] carried out high-throughput RNA sequencing on the myocardium of a mouse model in order to identify circRNA expression. They determined that 58 circRNAs were markedly differentially expressed. Among them, 29 circRNAs were downregulated, whereas 29 circRNAs were upregulated. Six overexpressed circRNAs (mmu_circ_0001697, mmu_circ_0001160, novel_circ_0008273, novel_circ_0009344, mmu_circ_0001625, and mmu_circ_0000431) and seven downregulated circRNAs (mmu_circ_0000652, mmu_circ_0000058, mmu_circ_0001058, mmu_circ_0000680, novel_circ_0000824, mmu_circ_0000547, and novel_circ_0004285) were confirmed by RT-qPCR. They found that mmu_circ_0000652 and mmu_circ_0001058 interacted with miR-195 and miR-21, both of which had roles in the metabolism of diabetic cardiomyopathy. Additionally, the downregulation of mmu_circ_0000652 was indirectly associated with the inhibition of BCL2 and stimulated apoptosis. The authors hypothesized that mmu_circ_0001160 might produce a protein linked to its host gene, ZNT7 (Zn^2+^ transporter 7), and participate in the early stage of diabetic cardiomyopathy. Overall, their results suggested that the aforementioned circRNAs could be potential diagnostic biomarkers in the early stage of diabetic cardiomyopathy (Table 3).

## 4. Ischemic Cardiomyopathy (ICM)

ICM is a common secondary cardiomyopathy and a major cause of heart failure and cardiac-related mortality worldwide [108]. It is a complex disease with interactions between environmental and genetic factors, including inflammation, microvessel dysfunction, apoptosis activation, and Ca^2+^ homeostasis disruption [109,110]. In the ischemic heart, many fetal and immediate-early genes are deregulated [111].

### Circ-Fndc3b

Circ-Fndc3b is a novel circRNA originating from exons 2 and 3 of the *Fndc3b* gene, harbored in chromosome 3, and it is mainly enriched in the cytoplasm [112].

Recently, Garikipati et al. [113] reported that circ-Fndc3b expression was downregulated in post-myocardial infarction mouse hearts and the cardiac tissues of patients with ICM. They also revealed that circ-Fndc3b did not serve as an miRNA sponge in vitro or in vivo. In addition, circ-Fndc3b regulated vascular endothelial growth factor (VEGF) expression and signaling by binding to RBPs fused in the sarcoma (FUS) and decreasing its level. Furthermore, the overexpression of circ-Fndc3b regulated the function of endothelial cells, diminished apoptosis in CMs in vitro, augmented angiogenesis, restricted the size of the infarct, maintained cardiac function and integrity of post-myocardial infarction, and mediated cardiac repair. The authors concluded that the upregulation of circ-Fndc3b might potentially serve as a new feasible therapeutic target to restrict ischemic injury.

## 5. Doxorubicin-Induced Cardiomyopathy (DIC)

Anthracyclines are the most potent anticancer chemotherapy drugs ever created and are used to treat a wide range of human neoplasms, including breast cancer, leukemia, malignant lymphomas, and sarcomas [114,115]. 

Doxorubicin (DOX) is one of the most effective types of anthracyclines developed since the 1960s [114,116,117]. Despite the highly advantageous anticancer effect of DOX, however, its clinical utility is limited by cardiotoxicity. Exposure higher than a threshold dose of DOX is associated with elevated risks of progressive heart failure and irreversible cardiomyopathies [118,119,120,121,122]. Genetic combinations are greatly involved in variable threshold doses of DOX, leading to DIC among individual patients [115]. Several hypotheses have been suggested, with activated reactive oxygen species (ROS) [117,123,124,125], topoisomerase II-β (TOP2β) inhibition [126,127], calcium overloading, and mitochondrial dysfunction [120,128] considered the potential mechanisms underlying DIC.

### 5.1. Circ-Amotl

CircRNA derived from angiomotin-like 1 (Circ-Amotl1),generated from exon 3 of the *angiomotin-like 1* (*Amotl1*) gene, is located in chromosome 11q21, and is a member of the Motin family. Amotl1, in cooperation with angiomotin (Amot) and angiomotin-like 2 (Amotl2), plays a key role in modulating the migration and polarity of endothelial cells [40,129,130,131].

In the neonatal heart compared with the mature heart, circ-Amotl1 is highly expressed, resulting in augmented CM function. This circRNA is found mainly in the nucleus and does not act as an miRNA sponge. A recent study showed that circ-Amotl1 expression conferred a protective effect (act) against DIC by promoting the activation of protein kinase B (PKB), also known as “AKT”, and the translocation of the nucleus [132]. Commonly, AKT is located in the cytosol and is inactive [133]. AKT is activated by phosphorylation and becomes pAKT, which is translocated to the nucleus, and through direct phosphorylation regulates proliferation-related factors in a positive manner and regulates the expression of pro-apoptotic proteins in a negative manner [134]. Circ-Amotl1 motivates AKT phosphorylation and pAKT nuclear translocation by binding AKT and PDK, leading to increased cell proliferation, survival, and cardioprotection in DIC.

Furthermore, the in vivo delivery of circ-Amotl1 could serve as a potential therapeutic target for prohibiting adverse cardiac remodeling [132]. 

### 5.2. Circ-FoxO3

The *forkhead box O3* (*FOXO3*) gene encodes both circ-FoxO3 and linear *FOXO3* (*FOXO3* mRNA). It is a transcription factor belonging to the forkhead family, which is distinguished by a forkhead DNA-binding domain [135,136,137]. 

The majority of FoxO3 proteins are situated in the cytoplasm and form a scaffold to bind to various RBPs [138]. FoxO3 is a crucial regulator in the insulin/insulin-like growth factor-1 signaling pathway and is related to apoptosis and cell death [137,139]. 

Du et al. [140] reported that circ-FoxO3 was upregulated in the heart tissue of DIC mice. They also found that the expression level of circ-FoxO3 was correlated with the tissue apoptosis level, left ventricular chamber dilation, and cardiac fibrosis, exacerbating DIC. 

Further, the in vivo delivery of siRNA-targeting endogenous circ-FoxO3 is regarded as a potential therapeutic approach to protecting myocardial cells by abrogating the effect of DOX.

### 5.3. Circ-ITCH

CircRNA-itchy E3 ubiquitin-protein ligase (Circ-ITCH, hsa_circ_0001141), which emanates from exon 7 to exon 14 of the *itchy E3 ubiquitin-protein ligase* (*ITCH*) gene, was first introduced by Memczak et al. [63] in 2013. Some studies have demonstrated that the circRNAs of *ITCH* are enriched in the human heart and human-induced pluripotent stem-cell-derived CMs (hiPSC-CMs) [75,79].

Circ-ITCH, mainly localized in the cytoplasm of hiPSC-CMs, sponges miR-330-5p. It is significantly increased in DIC and aggravates DOX-induced cardiac injury. Conversely, circ-ITCH expression is decreased in the heart tissue of patients with DIC, and the overexpression of circ-ITCH confers protection against DIC by sponging miR-330-5p and upregulating SIRT6, survivin, and SERCA2a. Thereby, circ-ITCH might be a novel therapeutic target for DIC (Table 4) [141].

## 6. Cardiomyopathy Caused by Alcohol 

One of the common causes of cardiomyopathy and heart failure is alcohol [142]. The term “alcoholic cardiomyopathy” is defined as a specific heart muscle disease found in individuals with excessive levels of alcohol consumption. Several mechanisms in alcoholic cardiomyopathy may correlate with detrimental cellular and structural changes to the myocardium, including oxidative stress, apoptotic cell death, and impaired mitochondrial bioenergetics/stress [143].

Yang et al. [144] performed a microarray assay using left ventricular tissues from three alcoholic cardiomyopathy samples and three controls in a mouse model to detect circRNA involvement in alcoholic cardiomyopathy, and found 643 circRNAs expressed in the left ventricular myocardium. Among them, 114 circRNAs were upregulated (viz., mmu_circ_011978, mmu_circ_011979, mmu_circ_011977, and mmu_circ_011982), while 151 circRNAs were downregulated (viz., mmu_circ_011976, mmu_circ_011975, mmu_circ_011981, mmu_circ_011980, and mmu_circ_011983). In their investigation, the bioinformatics analysis revealed that each circRNA could bind to more than five different miRNAs. Further, the qRT-PCR validation showed a reduced expression level in only one circRNA (viz, circRNA_011975), and this finding was subsequently corroborated by the microarray analysis. The expression of another two circRNAs was inconsistent with the microarray results.

## 7. CircRNAs in the Animal Model of Cardiomyopathy

There is a paucity of research into the functional role of circRNAs in cardiomyopathies in animal models. A previous investigation reported that circRNAs originating from the *titin* gene, usually with complicated exon structures, are involved in heart disease development [84]. In a study of the *RBM20* knockout mice heart, no TTN I circRNA expression was generated [84]. A prior study on the expression profile of the mice heart reported that the differential expression of circSLC8A1, the most abundant circRNA in CMs, could act as an endogenous sponge for miR-133a and regulate the expression of miR-133a targets (serum response factor (Srf), connective tissue growth factor (Ctgf), adrenoceptor beta 1 (Adr β 1), and adenylate cyclase 6 (Adcy6)) in cardiac hypertrophy in vivo [28]. Wang et al. [145] concluded that mitochondrial fission and apoptosis-related circRNA (MFACR) had pathogenic roles in the ischemic heart. They also reported that MFACR regulated mitochondrial fission and apoptosis in the heart by sequestering miR-652-3p. Moreover, in their study, miR-652-3p directly lessened mitochondrial protein 18 kDa (MTP18) and, thus, attenuated mitochondrial fission, CM apoptosis, and myocardial infarction in in vitro and in vivo models [145]. 

## 8. Conclusions

CircRNAs comprise an abundant, diverse, stable, and conserved class of regulatory RNA molecules that may represent a new type of diagnostic or prognostic biomarker of cardiac diseases given the limitations in the existing diagnostic markers. Nevertheless, our knowledge of the expression patterns of circRNAs is still in its nascent stages. Indeed, research is warranted into the identification of circRNAs and their localization and degradation, as well as their biological and pathophysiological roles and potential use for therapeutic or diagnostic purposes. Investigations have already been undertaken to discover the roles that circRNAs play; still, many obstacles remain to be overcome. By way of example, some genes such as the human CACR do not have a homologous gene in mice. Therefore, investigations in this domain are limited to cell lines (in vitro). Furthermore, not only is the number of patients involved in studies limited due to the low participation rate of patients in genetic testing, but also the clinical collection of cardiac tissues from patients poses a major challenge, undermining validation. Future expression research needs to feature appropriate endogenous control for data normalization. In this regard, several recent studies have probed into the roles of circRNAs as miRNA sponges, RBP holders, and parental gene expression regulators in physiological and pathophysiological states. In light of the evidence accumulated thus far, circRNAs could be considered novel diagnostic or prognostic biomarkers and therapeutic targets in diseases, including cardiomyopathies. Still, further in-depth functional studies are needed in this new field.

## Figures and Tables

**Table 1:** The circular RNAs involved in dilated cardiomyopathy.

Circular RNAs	Related Disease	Expression	Methods	Samples	Ref
Circ-SLC8A1	DCM	Up	RNA high-throughput sequencing and qRT-PCR	Heart samples	[78]
circ-SLC8A1 circ-CHD7 circ-ATXN10	DCM	Up	RNA sequencing and qRT-PCR	Heart samples	[79]
Circ-DNA6JC	DCM	Down	RNA sequencing and qRT-PCR	Heart samples	[79]
circTTN_70 circTTN_132 circTTN_34 circTTN_52 circRYR2_71 circRYR2_95	DCM	Down	Read-through circRNA	Heart samples	[80]
circSLC8A1_11 circ-SLC8A1_12	DCM	Up	Read-through circRNA	Heart samples	[80]
circ- EBF1 circ- ZNF670 circ- NBEA	DCM	Down	RNA high-throughput sequencing and qRT-PCR	Heart samples	[81]
circ- FAT1 circ- ICA1 circ- LYPLAL1	DCM	UP	RNA high-throughput sequencing and qRT-PCR	Heart samples	[81]
circ- MYH7 circ- SEC23A circ- TTN circ- PDE1C circ- CTNND2 circ- ATRX and OR2A1-AS1	DCM	Down	RNA high-throughput sequencing	Heart samples	[81]
circ- TTN circ- BTBD7 circ- NHLRC2 circ-DHX40 circ- G083903 circ- PKN2	DCM	UP	RNA high-throughput sequencing	Heart samples	[81]

**Table 2:** The circular RNAs involved in hypertrophic cardiomyopathy.

Circular RNAs	Related Disease	Expression	Methods	Samples	Ref
circDNAJC6 circMBOAT2 circTMEM56	HCM	Down	qRT-PCR	Serum samples	[92]
hsa_circ_0043762 hsa_circ_0036248 hsa_circ_0071269	HCM	-	Microarray	Plasma samples	[93]
HRCR	Cardiac hypertrophy and heart failure	Down	Microarray and qRT-PCR	Animals model	[94]

**Table 3:** The circular RNAs involved in diabetic cardiomyopathy.

Circular RNAs	Related Disease	Expression	Methods	Samples	Ref
circHIPK3	Diabetic cardiomyopathy	Up	qRT-PCR	Animals model	[102]
CACR	Diabetic cardiomyopathy	Up	qRT-PCR	Serum samples and cell culture	[103]
circRNA_000203	Diabetic cardiomyopathy	Up	CircRNA microarray and qRT-PCR	Animals model	[104]
circRNA_010567	Diabetic cardiomyopathy	Up	CircRNA microarray and qRT-PCR	Animals model	[105]
mmu_circ_0001697 mmu_circ_0001160 novel_circ_0008273 novel_circ_0009344 mmu_circ_0001625 mmu_circ_0000431	Diabetic cardiomyopathy	Up	RNA sequencing and qRT-PCR	Animals model	[106]
mmu_circ_0000652 mmu_circ_0000058 mmu_circ_0001058 mmu_circ_0000680 novel_circ_0000824 mmu_circ_0000547 novel_circ_0004285	Diabetic cardiomyopathy	Down	RNA sequencing and qRT-PCR	Animals model	[106]

**Table 4:** The circular RNAs in doxorubicin-induced cardiomyopathy.

Circular RNAs	Related Disease	Expression	Methods	Samples	Ref
circ-Amotl1	DIC	Up	Microarray and qRT-PCR	Human cardiac tissues and Animals model	[130]
circ-Foxo3	DIC	Up	Circular RNA sequencing and qRT-PCR	Animals model	[138]
CircITCH	DIC	Down	qRT-PCR	hiPSC-CMs and heart samples and animals model	[139]

## Data Availability

Not applicable.

## Abbreviations

circRNAs	Circular RNAs
DCM	Dilated cardiomyopathy
HCM	Hypertrophic cardiomyopathy
RCM	Restrictive cardiomyopathy
ARVCM	Arrhythmogenic right ventricular cardiomyopathy
ncRNA	Noncoding RNA
piRNAs	Piwi-interacting RNAs
miRNAs	MicroRNAs
siRNAs	Small interfering RNAs
lncRNAs	Long noncoding RNAs
rRNAs	Ribosomal RNAs
tRNAs	Transfer RNAs
snRNAs	Small nuclear RNAs
snoRNAs	Small nucleolar RNAs
ecircRNA	Exonic circular RNAs
RCMs	Reverse complementary matches
ADAR	Adenosine deaminases acting on RNA
ciRNAs	Circular intronic RNAs
elciRNAs	Exon-intron circular RNAs
HUGO	Human genome organization
RBPs	RNA-binding proteins
Hectd1	HECT domain E3 ubiquitin-protein ligase 1
Ppp2r3 α	Protein phosphatase 2 regulatory subunit B’’ alpha
Slc8a1	Solute carrier family 8 (SODIUM-CALCIUM EXCHANGER) member A1
Dmd	Dystrophin
Ttn	Titin
CMs	Cardiomyocytes
ARID1A	AT-rich interaction domain 1A
FNDC3B	Fibronectin type III domain containing 3B
CACNA1D	Calcium voltage-gated channel subunit alpha1 D
SPHKAP	SPHK1 interactor, AKAP domain containing
ALPK2	Alpha kinase 2
AASS	Aminoadipate-semialdehyde synthase
FIRRE	Firre intergenic repeating RNA element
TMEFF1	Transmembrane protein with EGF-like and two follistatin-like domains 1
CHD7	Chromodomain helicase DNA-binding protein 7
ATXN10	Ataxin 10
DNAJ6C	DnaJ heat shock protein family (Hsp40) member B6
ICA1	Islet cell autoantigen 1
BTBD7	BTB domain containing 7
FAT1	FAT atypical cadherin 1
LYPLAL1	Lysophospholipase-like 1
NHLRC2	NHL repeat containing 2
DHX40	DEAH-box helicase 40
PKN2	Protein kinase N2
MYH7	Myosin heavy chain 7
EBF1	EBF transcription factor 1
ZNF670	Zinc finger protein 670
SEC23A	SEC23 homolog A, COPII coat complex component
NBEA	Neurobeachin
PDE1C	Phosphodiesterase 1C
CTNND2	Catenin delta 2
ATRX	ATRX chromatin remodeler
OR2A1-AS1	OR2A1 antisense RNA 1
NPPA	Natriuretic peptide A
MYH6	Myosin heavy chain 6
RYR2	Ryanodine receptor 2
SCAF8	SR-related CTD associated factor 8
TIAM2	TIAM Rac1 associated GEF 2
RBM20	RNA-binding motif protein 20
MBOAT2	Membrane bound O-acyltransferase domain containing 2
TMEM56	Transmembrane protein 56 (TLCD4 (TLC Domain Containing 4))
HRCR	Heart-related circRNA
ARC	Activity-regulated cytoskeleton-associated protein
GO	Gene ontology
TRP	Transient receptor potential
HIPK3	Homeodomain interacting protein kinase 3
ceRNA	Competing endogenous RNA
COL1A1	Collagen type I alpha 1 chain
COL3A1	Collagen type III alpha 1 chain
CACR	Caspase-1-associated circRNA
Myo9a	Myosin IXA
COL1A2	Collagen type I alpha 2 chain
α-SMA	α-smooth muscle actin
CTGF	Connective tissue growth factor
TGF-β1	Transforming growth factor beta 1
BCL2	B-cell lymphoma 2
ZNT7	Zinc transporter 7 (SLC30A7)
Fndc3b	Fibronectin type III domain containing 3B
VEGF	Vascular endothelial growth factor
FUS	RNA-binding protein fused in the sarcoma
DIC	Doxorubicin-induced cardiomyopathy
DOX	Doxorubicin
ROS	Reactive oxygen species
TOP2β	Topoisomerase II-β
Amotl1	Angiomotin-like 1
Amotl2	Angiomotin-like 2
AKT	Protein kinase B (PKB)
pAKT	Phosphorylated AKT
PDK	Pyruvate dehydrogenase kinase
FOXO3	Forkhead box O3
RBPs	RNA-binding proteins
ITCH	Itchy E3 ubiquitin-protein ligase
hiPSC-CMs	Human-induced pluripotent stem-cell-derived cardiomyocytes
SIRT6	Sirtuin 6
SERCA2a	Sarco/endoplasmic reticulum calcium (Ca^2+^) ATPase
ACM	Alcoholic cardiomyopathy
circ-ITCH	CircRNA-itchy E3 ubiquitin-protein ligase
qRT-PCR	Quantitative reverse transcription-polymerase chain reaction
rt-circRNAs	Read-through circRNAs
